# Quality and impact of appraisal for revalidation: the perceptions of London’s responsible officers and their appraisers

**DOI:** 10.1186/s12909-015-0438-7

**Published:** 2015-09-21

**Authors:** Ann Griffin, Daniel S. Furmedge, Deborah Gill, Catherine O’Keeffe, Anju Verma, Laura-Jane Smith, Lorraine Noble, Ray Field, Celia Ingham Clark

**Affiliations:** 1 University College London Medical School, 74 Huntley Street, London, WC1E 6 AU UK; 2 Health Education North West London, London, UK; 3 NHS England, London, UK

**Keywords:** Revalidation, Appraisal, Quality, Clinical governance, Management

## Abstract

**Background:**

To evaluate NHS England London region’s approach to the revalidation appraisal of responsible officers in London, exploring perceptions of the quality and impact of the appraisal process. Revalidation is the process which aims to ensure doctors in the UK are up-to-date and fit to practice medicine thus improving the quality of patient care. Revalidation recommendations are largely premised on the documentation included in annual appraisals, which includes the professional development a doctor has undertaken and supporting information about their practice.

**Methods:**

A pan-London qualitative study exploring the views of responsible officers and their appraisers about the revalidation appraisal process. The study aimed to gain an in-depth understanding of the experiences and perceptions of the participants. Responsible officers were purposefully sampled to represent the broadest range of designated bodies. Data analysis generated themes pertaining to quality and impact of appraisal for revalidation with the potential to feed into and shape the evolving system under investigation.

**Results:**

The central importance of highly skilled appraisers was highlighted. Both groups reported educational opportunities embedded within the appraisal process. Independent appraisers, not matched by clinical speciality or place of work, were considered to take a more objective view of a responsible officer’s practice by providing an ‘outsider perspective’. However, covering the breadth of roles, in sufficient depth, was challenging. Participants reported a bias favouring the appraisal of the responsible officer role above others including clinical work. Appraisal and revalidation was perceived to have the potential to improve the healthcare standards and support both personal development and institutional quality improvement.

**Conclusions:**

Responsible officers play a central role in the revalidation process. Getting responsible officer appraisal right is central to supporting those individuals to in turn support doctors and healthcare organisations in continuous quality improvement. The complexity and importance of the role of responsible officer may make achieving an appraisal of all roles of such individuals problematic. This evaluation suggests responsible officer appraisal was perceived as educational and effective.

## Background

Public, patient and governmental concerns about the governance of the medical profession have resulted in a shift from permissive professional autonomy to regulated professional accountability. Ensuring doctors are fit to practise has become integral to the wider discourse about patient safety and quality care, and increasing public confidence in the medical profession has been a dominant driver in the introduction of revalidation. Revalidation began in December 2012 and marked a significant shift in the professional regulation of doctors. Revalidation is the process by which doctors in the United Kingdom (UK) are assessed against the General Medical Council’s (GMC) standards, *Good Medical Practice* [1]. If successful the GMC grants doctors a five-yearly licence to practise medicine. Revalidation is put forward as the process by which patients, the public, employers and other healthcare professionals will be ‘assured’ that licensed doctors are up-to-date in their skills and knowledge and practise in accordance with the appropriate professional standards [2]. Whilst broadly supported by the profession [3–5] and regarded as a mechanism which will improve performance by involving all doctors in a common process of governance, evidence for a causal relationship between revalidation and improved quality is at present limited [6–9].

Revalidation requires doctors to pro-actively demonstrate that they are up-to-date and fit to practise. This involves doctor’s engaging in annual appraisal and providing a body of supporting evidence, in keeping with the GMC’s Good Medical Practice framework for appraisal and revalidation [2]. The supporting information required for revalidation includes five yearly multi-source feedback from colleagues and patients and a quality improvement activity. Each annual appraisal requires supporting information that shows progress against last year’s personal development plan, typically 50 h of continuous professional development (CPD), any significant events that the doctor has been involved with, two reviews of clinical cases, a list of compliments and complaints and reflective writing demonstrating how these activities have impacted on a doctor’s practice [10].

Responsible officers (ROs), doctors with a new statutory role in UK healthcare, make recommendations to the GMC about an individual doctor’s fitness to practice based on the appraisal portfolio and appraiser’s statements. More broadly, RO’s oversee the co-ordination, delivery and quality assurance of appraisal and revalidation for all doctors that they have a ‘prescribed connection’ or formal relationship with. Typically but not uniformly, they are medical directors of healthcare organisations and are also accountable for clinical governance systems within these. By linking clinical governance and professional regulation, RO’s have an opportunity to enhance patient safety and quality improvement by maintaining professional standards and promptly identifying doctors with performance issues [11]. As senior clinicians and GMC-registered doctors, ROs were amongst the first to undergo revalidation based on annual appraisal.

**Fig. 1:**
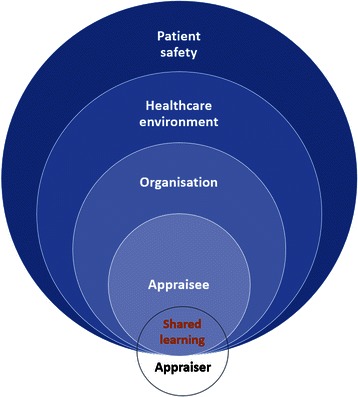
The responsible officer network in London

In England, there are two categories of RO (Fig. 1): level 1 ROs include those responsible for a designated healthcare organisation and the revalidation recommendation of doctors within it and ROs in Area Teams who are responsible for revalidation recommendations on all GPs in their area. Higher level ROs include four regional medical directors in London, each responsible for making revalidation recommendations on all level 1 ROs in their region.

Appraisal is the bedrock of the revalidation process. It sits within the field of self-regulatory professional development and aims to improve clinical care by encouraging doctors to tailor their CPD in ways that positively impact on their professional performance. Evidence suggests that CPD could improve quality of care [6, 12, 13] although appraisal has been heavily criticised for variable consistency and robustness [14]. To address this, appraisal has become increasingly formalised and structured. To meet revalidation requirements doctors must provide a core set of supporting information and there is increasing emphasis on the quality of the appraisal interview and its outputs: the summary of discussion and the Personal Development Plan (PDP). The appraisal interview itself is vital for effective appraisal [15–17]. Poorly trained appraisers give inadequate feedback and over-estimate the evidence and skills of their appraisees [15] and the benefits of appraisal can be undermined if there is ineffective organisational support [18].

Revalidation is already affecting appraisal completion rates and quality with overall rates of appraisal of UK doctors increasing from 63 % in 2010–11 to 76 % in 2012–13 [19]. The Revalidation Support Team report, *The early benefits and impact of medical revalidation* [5], reported an increased quality of appraisal outputs since revalidation but that concerns exist regarding the relevance of appraisal, particularly its rather procedural approach and focus on compliance with process.

This qualitative evaluation explored the views of ROs and their appraisers regarding the RO appraisal process and practice in NHS England London region. Using a lens of ‘quality’ this study builds on existing research about RO perceptions of revalidation and focused on perceptions of effectiveness, robustness, and perceived impact as well as the acceptability of this activity as a way of both challenging and supporting ROs in their roles [20]. This study aimed to explore the perceptions of quality involved in RO appraisal from the perspective of the RO as well as their appraisers and to inform the revalidation community (doctors, their appraisers, ROs and medical directors) as policy and practice are shaped and embedded.

## Methods

### Design

A qualitative study, using both focus group and one-to-one interviews, was carried out with the aim of gathering in-depth perspectives on the quality of the RO appraisal process in terms of effectiveness, robustness, perceived impact and acceptability.

### Setting

NHS England London region in 2013 after the first round of RO revalidation appraisal.

### Participants

All 24 RO appraisers in NHS England London region were invited to participate and 17 took part in the study. There were 140 ROs in NHS England London region at the time of the study. These ROs were acknowledged as a heterogeneous population in terms of their experience of appraisal, experience of managerial or strategic level support of appraisal and other quality enhancement activities [21]. They also represented a very diverse range of organisations and groupings although all had access to standardised training (including a series of group training sessions) for this role from NHS London. Using an organisational typology this group was purposefully sampled to represent this diversity sampling the full range of type, size and focus of designated bodies. Participants were invited based on their organisational classification with the aim of ensuring at least three ROs from each classification. The aim was to sample 10–15 % of the total RO cohort (Table 1). This sample grew iteratively during data collection as it became clear that small independent sector organisations were the most diverse and would therefore need more intensive sampling to capture the fullest range of perspectives. A total of 28 ROs participated (20 %) and from the entire range of organisational classifications (see column 4 Table 1).

**Table 1:** Organisation classification of the designated bodies and RO sample stratification

Organisation classification	Number of ROs	Number of ROs invited	Number of participants interviewed
NHS Foundation Trust	16	6	4
NHS Trust	27	9	6
NHS Area Teams (for general practice)	3	3	3
Independent – Large^a^	26	4	4
Independent – Small^b^	43	18	7
Independent – Other^c^	26	7	4
Total	140	47	28

^a^ >50 employees

^b^ < 50 employees

^c^ Includes charities and third sector organisations

### Intervention

RO appraisers were selected through an open recruitment process and required essential attributes [22]. NHS England London region ensured that RO appraisers represented the diversity of the RO population in terms of:

- organisational affiliation
- geographic location
- ethnic and gender distribution
- clinical speciality

RO appraisers were matched to ROs to ensure there was no co-location or conflict of interest in order to facilitate an appraisal that was as objective and independent as possible. They were not matched for clinical speciality but RO appraisers were asked their preferences about appraising different clinical specialities. This specific recruitment of external or ‘outsider’ RO appraisers differs from many models of RO appraisal elsewhere in the UK at the time of this study.

The study was assessed as a service evaluation by UCL Joint Research & Ethics Committee Office.

### Data collection

Semi-structured interview schedules (for ROs) and focus group questions and prompts (for RO appraisers) were devised based on the evaluation questions identified above. Participants were invited by email. Participation was voluntary and written consent was obtained.

### Data collection from RO appraisers

A focus group approach was used with RO appraisers to capture the range of approaches and experiences and identify common ideas and factors associated with a satisfactory or unsatisfactory appraisal. Each group used both an experienced facilitator and an observer who made field notes. One RO appraiser could not attend a group and was interviewed individually.

### Data collection from ROs

ROs participated in one-to-one, semi-structured interviews at a location of their choice in person or via telephone. A one-to-one interview approach was used to ensure the focus remained on personal experiences and perceptions and to maximise participation in the study.

### Analysis

Focus groups and interviews were audio-taped and transcribed verbatim. Field notes were used to support the analysis. *QSR NVivo 10*© was used to manage and organise the data.

Data analysis was inductive and borrowed from a grounded theoretical approach, with meaning flowing from the data to form themes and concepts. It was also deductive and explored issues posed by the research question [23]. RO appraiser and RO data were initially analysed separately to explore each dataset independently before looking for commonality. Initial analysis was undertaken by four research staff independently (AG, DG, CO and DF). Tentative coding themes were discussed and refined. Once this framework reached saturation a final coding framework was agreed and applied to the entire data set. Inter-coder comparison for coding consistency using *NVivo 10* demonstrated a 98 % agreement between coders.

### Limitations

This was a small scale study set in London; however, the participants were sampled across the range of organisations so that the breadth of views could be explored. This study was participatory and individuals volunteered to take part. Those who did not respond to the email invitations may have more negative views about revalidation, appraisal and the approach taken in London and affected the results. Furthermore, the considerable number of non-responders in the request for participation from the ‘small independent’ sector may mean the participants from this group may not have been representative. Qualitative data and its analysis have their own biases. In order to help mitigate against this data was independently coded and inter-rater coding comparisons made to ensure agreement.

It should be recognised that this research reports on a very specific type of appraisal, of ROs. There is emerging evidence that their opinions of appraisal and revalidation are more positive than the wider population of UK doctors [5]. This study also took place in early 2013, when revalidation was only just beginning. At this stage responsible officers had not begun to make recommendations to the GMC about revalidating the doctors that they had prescribed connection with and many were setting up and refining systems for appraisal and clinical governance. This limits this papers ability to comment more authoritatively about the actual impact of revalidation on patient safety and quality improvement.

## Results

**Fig. 2:**
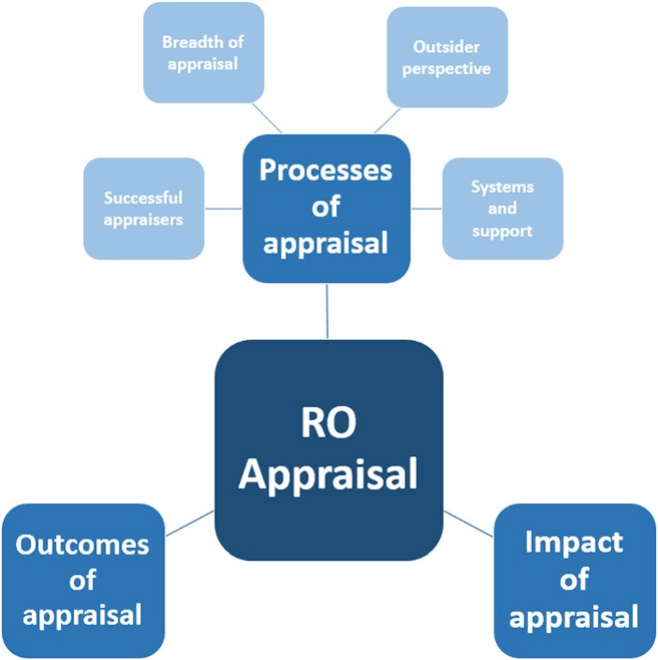
Themes identified from participants’ perceptions of RO appraisal

Three overarching themes were identified (Fig. 2).

1. **Perceptions of a good quality appraisal**

Participants noted several features of the appraisal which they felt contributed to its quality:

- **Successful appraisals**

An experienced and skilful appraiser was seen as central to a successful RO appraisal. ROs perceived appraiser preparedness as necessary for effective appraisal, particularly familiarity with appraisal requirements, advance correspondence and having reviewed supporting evidence in advance. RO appraisers also identified preparedness as central to a good appraisal, however they highlighted marked variation in individual RO readiness for appraisal and noted that work before the interview, to ‘get ROs ready’ was often considerably more demanding than anticipated:

*Because this is the first year we’ve all wanted to go beyond the call of duty and try and do the best we can to support, facilitate … we think it’s hard for them as well, some of them just don’t know what the process is. We’ve probably all of us been more helpful than we perhaps should have been or needed to be. I think for next year perhaps we need to contain it a little bit more.*

RO appraiser focus group 1

Successful appraisal required relationships. The long period of negotiation and feedback before the interview meant relationships, although potentially awkward due to their novelty and high stakes nature, were often established before the interview took place:

*You had to really use your best communication skills because you were having to form, you know a very close relationship with somebody and you didn’t know how happy or unhappy they were with that, and you didn’t know how much of their prejudice or attitude towards the whole validation process was going to influence how they responded to you.*

RO appraiser focus group 2

RO appraisers good interpersonal and communication skills were reported by ROs as key ingredients making appraisal successful. Being appropriately curious and stretching the RO led to supportive conversations which promoted reflection. ROs described the importance of allowing space to let them to talk and for ideas for their professional development to ‘fall out of the conversation’. Skilled questioning challenged and made appraisees stop, think and reflect on practice in a way that sometimes they had not been able to do before. ROs used words like “therapeutic”, providing an opportunity to “step back and take stock, as it were”:

*It was fantastic, because actually it wasn’t you know ‘Oh tell me about this case’ - and the nitty gritty of stuff that I do on a day to day, it was more about my style of medicine, my management style, my impact, my highs, my lows, my strengths, my weaknesses … so I suppose more like a general appraisal … and actually I found it so much more revealing perhaps and satisfying than one I’d had previously. I mean not that the others were bad, but it was all … you felt that people were ticking boxes. Whereas this was actually ‘God, I have to think about this’*

RO1

b. **Breadth of the appraisal**

Many ROs described working in several roles: as a clinician, as a medical director, private or other work, and some found it difficult to provide supporting information to fully bring out the complexities of each role. Mostly, ROs felt that the appraisal did cover the breadth of their roles through a systematic approach. However, there was a general view that RO appraisal was primarily concerned with the RO role, concentrating on RO competences. This left a gap, particularly when appraising clinical work.

*But the other bit of appraisal which is you know where are you going in your practice, what’s difficult, what’s easy … you know how should you develop it … what else are you going to do with your clinical career and all this sort of stuff – it wasn’t really part of that … but it is supposed to be part of appraisal.*

RO19

Some RO appraisers found getting the balance between evaluating supporting information and ensuring standards were met challenging. There was a tension in some instances to provide reams of supporting information, quantity over quality. ROs also expressed a concern about which supporting evidence was the best to present.

*The difficulty for me was rather more what you didn’t put in rather than what you put in. I mean, no appraiser looked at from their standpoint is going to want to wade through mountains of paperwork.*

R011

Some RO appraisers noticed a lack of pre-appraisal reflection, suggesting many ROs used the appraisal interview as their main vehicle for reflective practice.

c. **The outsider perspective**

Whilst there were a range of views about being appraised by somebody who was in effect ‘an outsider’, either to their organisation or their clinical specialty, largely ROs reported that this perspective was useful for a range of reasons. Different outlooks and insights were deemed helpful, in terms of lack of prior assumptions and enhanced questioning. Additionally, having no prior connection offered freedom to discuss sensitive issues:

*I think the fact that it’s independent is really good because … for example all my appraisals to date have been done by my friend who’s also the medical director who’s also a head of department. And some of the issues, even patient safety issues, may be to do with the way he runs the department…You might want to say something confidential about your health that you don’t want your colleagues to know about, so I think having an independent person is really good.*

RO27

However, a small number of ROs felt that the outsider perspective was less valuable particularly when appraising clinical aspects of their role. Four ROs suggested that a non-clinically matched appraiser could not assess clinical supporting information in the same way that a speciality-matched appraiser might:

*No… I don’t think it did a good job there. That whole conversation never really took place… the appraiser wasn’t within my field, so I wouldn’t expect them to know or understand that at the end of the day.*

RO10

RO appraisers agreed with the majority of ROs that there were important benefits from not being in the same clinical specialty:

*There’s a fundamental issue …. Whether or not you should be a specialist in the area of the doctor … and I’ve got very strong views that we don’t to have to be.*

RO appraiser focus group 2

RO appraisers did not seem troubled by any perceived power imbalance when appraising these very senior doctors:

*I felt that certainly for you know some medical directors of big Trusts, you know, that actually they were coming to me to be appraised… so I was then in the driving seat, as opposed to the other way round, so I had the psychological upper hand*.

RO appraiser focus group 2

d. **Systems and support**

ROs and RO appraisers expressed the importance of organisations in providing clear guidance, support networks and robust systems, including a capable IT system for appraisal.

*Well the organisation has actually said, well this is something that needs to be done, let’s go for it.*

RO16

Having realised the significance of these elements, ROs reflected on improving similar processes within their own organisations.

*I’m beginning to realise that actually this process could be made better. So we’re having external auditors come in and look at the appraisal process again with a view to possibly seeing if they could be improved further.*

RO13

2. **Perceptions of outcomes of the revalidation appraisal**

The RO appraisal was viewed as an educational event in itself. ROs reported learning to reflect but also about setting up appraisal processes, learning appraisal skills, learning how to be an RO and planning their own learning. Several ROs revealed how their appraisal had made them re-examine systems in their organisation. They discussed improving pre-existing appraisal systems and the supply of supporting information, allocating ‘independent’ appraisers, effective technological support, and quality assurance of the process.

Learning about the RO role was a product of the appraisal preparation and interview. Appraisers and appraisees shared tips based on personal experiences, and RO appraisers disseminated good ideas from other ROs, heard through appraisal conversations:

*…the forcing into looking quite deeply at all areas of the job, and particularly the sort of explicit confrontation of the fact that you know being RO you are responsible for a lot of stuff around the organisation and understanding what that was, your responsibilities … you know showing this to the board and evidencing all that etc. etc. was a wake-up call.*

RO26

Most ROs felt that they had produced a useful PDP that reflected the diversity of their roles, but was targeted towards priority areas for their own development.

RO appraisers felt their role went beyond appraiser to that of mentor, supporter and teacher. This was felt to be particularly important in those organisations where the RO was poorly supported and for ROs who were not thought to meet the expected standard.

3. **Perceptions of the impact of the revalidation appraisal**

Undergoing revalidation appraisal made ROs consider the role of appraisal in general. They reported it to be driving institutional culture change and raising standards. Many ROs felt that their role and revalidation more generally, facilitated organisational quality improvement and was important for patient safety:

*It’s one of the most important developments that we’ve had … it’s about protecting the public. And so I see it as a fundamental role that we must do.*

RO12

They felt it would improve standards by encouraging doctors to think about their work and plan their own learning:

*…there may well be some doubters and defaulters and people are always going to negative about it, but I think it is a mechanism to improve quality and give doctors the opportunity to actually …help to look at the work that they do and to you know look at their PDPs and move things forward in a positive way, so I think it’s a good thing.*

RO21

RO appraisers agreed, talking about their role in terms of as the ‘enforcer of standards’ and described ways in which both the process and their expectations were raising standards.

In non-NHS organisations, revalidation and an RO were additionally seen as ‘quality markers’. The whole process brought them ‘back into the loop’, uniting them with NHS doctors in a common governance process and thus into a wider a medical community:

*I still felt that for the organisation it was important to have somebody who was a designated responsible officer, and also who understood the processes. Because if there was a problem with an NHS doctor, or a doctor whose primary responsible officer was in the NHS, I would still have to liaise with them, and in fact I’ve had to do that in the past with somebody else who had worked with us then left then got reported to the GMC and I was involved in the kind of … you know the reporting and the supervision of that individual… Having had that experience I didn’t … I didn’t want us to be an organisation that was outside the loop.*

RO27

## Discussion

### Summary of findings

Participants reported that RO revalidation appraisals were acceptable and of high quality. Quality resulted from a range of factors, but was dependent on the RO appraisers’ commitment to make it work, to form relationships, to support ROs before appraisal, and to have meaningful and skilful appraisal conversations. RO appraisers recognised the importance of the skills they already possessed from previous experience as appraisers, particularly the importance of interpersonal skills and the ability to take a systematic approach. These skills were also recognised by ROs as important factors supporting effective appraisal. ROs reported revalidation appraisal as more challenging than previous appraisals, by which they typically meant that it was more probing and thought-provoking, in part due to being appraised by ‘an outsider’. The importance of effective systems, support, guidance and feedback were noted.

Both RO appraisers and ROs recognised learning opportunities embedded in the appraisal conversation. RO appraisers recognised their role as mentor, teacher and distributor of good practice. ROs mentioned learning about the RO role as a direct result of engagement with the appraisal: particularly learning to improve their own appraisal skills, learning to set up appraisal systems and defining educational objectives for personal development.

**Fig. 3:**
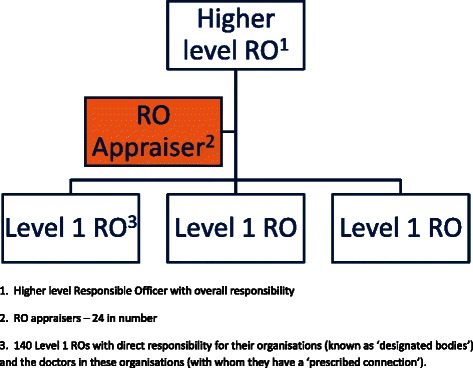
The wider benefit of appraisal

Beyond personal development and shared learning, revalidation appraisal was perceived to foster positive cultural shift, quality improvement and process development at an institutional level, bringing UK registered doctors into one medical community and raising standards to improve patient care. This wider benefit of the appraisal is conceptualised in Fig. 3.

### What makes good quality appraisal for ROs?

At the heart of effective appraisal is the appraiser [15–17]. This study evaluated a group of highly skilled, experienced appraisers, motivated to perform this innovative appraisal activity. ROs had a high degree of satisfaction, with only one reporting a negative experience. These findings counter the discourse about appraisal variability [24] and corroborate the view that appraisers require effective communication and interpersonal skills [15, 25, 26]. Appraisers talked about using communication skills to form relationships with their ROs, and appeared to possess ‘advanced skills’ [25] as they successfully engaged with senior doctors. Unlike the appraisal literature outside medicine, ROs did not seem to find probing or challenging questions threatening [15], instead finding this constructive and promoting reflection.

It seemed important to RO appraisers to form relationships with their appraisees. This may be because of their unfamiliarity with these senior doctors and their organisations, and their concerns about whether ROs had positive views about appraisal and revalidation. Senior colleagues, ‘high flyers’ are a challenging group to appraise [27]. The status of doctors may influence the appraisal [28], with appraisers concerned about discussing performance issues [29]. This is mirrored outside medicine, where managerial reluctance to make negative judgments about an individual’s performance has been attributed to manager fear about demotivation, accusations of lack of managerial support and lack of training in how to simultaneously maintain performance judgment roles with being an understanding counsellor [30, 31]. RO appraisers felt that forming relationships mitigated against discontentment with appraisal. ROs did not report feeling outranked in the appraisal process, possibly as this process was different to appraisals where there is usually a superior-subordinate relationship [32]. This power relationship is perhaps less evident due to the seniority of the ROs being appraised.

RO appraisers engaged in considerable pre-appraisal contact with ROs. This ‘pre-interview phase’ sets the scene for the subsequent conversation but is largely neglected in the literature [15]. In this phase the agenda for the appraisal is discussed. In the present study, much of the discussion in this preparatory period was about supporting information. The high degree of preparation and systematic approach to appraisal was noted as significant by RO appraisers, reflecting views of appraisers in other evaluations [27]. Lengthier-than-anticipated appraisals were also noted in the Revalidation Support Team Report, likely due to this being the first round of revalidation appraisal [5].

### Appraisal as an educational event

A significant impact of RO revalidation appraisal was its potential as an educational event, a process for learning and sharing knowledge, which resulted in the personal development of both RO appraisers and ROs. ROs mentioned opportunities to reflect on practice during their appraisal. Given the emerging focus on reflective practice and its relation to revalidation appraisal [2], this positive view of the reflection effected by the RO appraisal is encouraging and significant. However, RO appraisers commented on the lack of written reflection in appraisal portfolios. The current evidence for reflective practice is theoretically persuasive but currently lacks an empirical basis and is an important area for further research [33].

As a result of the appraisal, ROs felt they were able to create a useful, achievable PDP. This supports previous findings in GPs and ROs who felt the appraisal process had enhanced their learning, improved their practice and encouraged them to engage in continuing professional development [34–36].

### The ‘outsider perspective’

ROs suggested that appraisal by an ‘outsider’, as was specific to this London model of revalidation appraisal was advantageous, with potential for a reflective, broader appraisal. Conversations explored roles from a new perspective and took a broader overview rather than trawling through specifics; this resulted in issues and learning falling out of appraisal dialogue. It was a significant advantage in relation to objectivity and facilitated discussions which involved organisational difficulties or patient safety concerns. This area is under-evaluated in the literature, although it is suggested that an appraisal should be done by someone who does not have line management responsibilities [2, 15]. Similarly, a study of surgeons from the USA discovered a strong preference for external assessment when re-licensure could not be issued on the basis of local appraisal alone although it must be noted that re-licensing processes in the USA differ significantly to those for current UK revalidation [37]. Appraisal by an ‘outsider’, rather than someone who is a line manager may avoid potential conflicts of interest and was suggested by some appraisees as a process that may facilitate whistleblowing and be a mechanism to support patient safety.

### Appraising breadth of roles

Appraisal is complex and requires a targeted approach [15]. The revalidation appraisal aspired to cover multiple competences across multiple roles but the present study highlighted a ‘responsible officer role bias’, defined as a focus on the role of the RO, sometimes at the expense of other roles held by the appraisee, particularly clinical work. This may have been due to this being a relatively small component of some of the RO’s overall workload. A minority of ROs strongly believed that this bias negated the central importance of their own clinical work and patient safety. The perceived difficulty in appraising a different clinical specialty has previously been identified [38]. Given the complexity of appraising multiple roles and following the considerable pre-appraisal preparation, this complex multiple-role appraisal may be over-ambitious. This has wider relevance with more doctors assuming ‘portfolio roles’ who, with revalidation will also need to be appraised for each role.

### Governance for all

‘Becoming part of the medical community’ was a view articulated by some ROs in the independent sector who felt that revalidation was a process that aligns doctors in private practice with their colleagues working in the NHS. Revalidation appraisal was described as driving up clinical standards and welcomed by most. This level of buy-in from senior healthcare staff may have contributed to the acceptability of the revalidation appraisal by those interviewed. ROs, as organisational managers seemed to be ‘early adopters’ of revalidation, acting as champions [5]. The importance of robust systems underpinned by a supportive culture was stressed, mirroring findings in another qualitative study of ROs in London [35].

Appraisal is relevant to every doctor, from trainee progression appraisals to revalidation. This study looked at the first round of the high-stakes appraisal of a specific group of high-achieving doctors, by experienced appraisers. For many doctors, particularly trainees and those outside of general practice, appraisal is sometimes undervalued and historically has had poorer engagement, although the roll out of revalidation is changing this [19]. This study also has relevance across healthcare with the introduction of revalidation in other professions such as nursing and pharmacy [39, 40].

## Conclusion

The views of appraisers and appraisees about the new process of revalidation appraisal highlighted key features of an effective appraisal. Participants noted the value of skilled and committed appraisers, the role of appraisal as a profound learning opportunity and the potential impact on patient safety and quality improvement. The results of this study from the first iteration of revalidation appraisal, whilst specific to this group carry significant messages for revalidation appraisal processes across healthcare disciplines.

## Abbreviations

CPD: continuing professional development
GMC: General Medical Council
GP: General Practitioner
NHS: National Heath Service
PDP: Professional Development Plan
RO: Responsible Officer
UCL: University College London
UK: United Kingdom
USA: United States of America
